# A meta-analysis of the proportion of antimicrobial resistant human *Salmonella* isolates in Ethiopia

**DOI:** 10.1186/2050-6511-15-51

**Published:** 2014-09-12

**Authors:** Getachew Tadesse

**Affiliations:** 1 Department of Biomedical Sciences, College of Veterinary Medicine and Agriculture, Addis Ababa University, P.O. Box 34, Debre Zeit, Ethiopia

**Keywords:** Antimicrobial resistance, Ethiopia, Humans, *Salmonella*

## Abstract

**Background:**

Antimicrobial resistant *Salmonella* is a global problem and recently, a strain on the verge of pan-resistance was reported. In Ethiopia, the therapeutic management of Salmonellosis is difficult because drug sensitivity tests are not routinely carried out and treatment alternatives are not available in most health care facilities. The objectives of this study were to estimate the temporal changes and proportions of drug resistant isolates in Ethiopia.

**Methods:**

Published studies on drug resistant *Salmonella* isolates were searched in Medline, Google Scholar and the lists of references of articles. Eligible studies were selected by using inclusion and exclusion criteria. Generic, methodological and statistical information were extracted from the eligible studies. The extracted data included the proportions of ampicillin, co-trimoxazole, chloramphenicol, ceftriaxone, ciprofloxacin and multi-drug resistant isolates. Pooled proportions were estimated by a random effects model.

**Results:**

The odds of multi-drug resistant isolates in the 2000s was higher than before the 1990s (OR =18.86, 95% CI = 13.08, 27.19). The pooled proportions of ampicillin, co-trimoxazole, chloramphenicol, ciprofloxacin and multi-drug resistant isolates in the 2000s were 86.01%, 68.01%, 62.08%, 3.61% and 79.56% respectively. *S*. Concord (>97%) was resistant to ampicillin, co-trimoxazole, chloramphenicol and ceftriaxone.

**Conclusion:**

The proportion of drug resistant isolates has increased since the 1970s. All drugs currently used for the treatment of Salmonellosis but ciprofloxacin are not reliable for an empirical therapy. Alternative drugs should be included in the essential drug list and measures should be taken to re-enforce the drug use policy.

## Background

The emergence of drug resistant pathogens is associated with a variety of biological, pharmacological and societal variables with the worst combinations in developing countries [1]. Antimicrobial resistant *Salmonella* is one of the global problems in present day clinical practices and recently, a strain on the verge of pan-resistance was reported [2]. In sub-Saharan Africa (SSA), the prevalence of MDR *Salmonella* has increased and outbreaks due to MDR strains were recorded [3-5]. Infections with MDR pathogens are associated with excess morbidity and mortality probably because of the co-selection of traits of drug resistance and virulence [6]. In developing countries like Ethiopia, the therapeutic management of the disease is difficult because drug sensitivity tests are not routinely carried out and treatment alternatives are not available in most health care facilities.

*Salmonella* is one of the major causes of gastroenteritis and fever in Ethiopia. The bacterium was isolated from a number of patients including pediatrics [7] and mal-nourished children [8]. *S*. Concord, *S*. Typhi, *S*. Typhimurium and *S*. Paratyphi were the dominant serotypes that accounted for 82.1% of the isolates from patients [9]. *S*. Concord was isolated from a bone processing plant [10], an immigrant in Ireland [11], diarrheal and/or febrile patients [12,13] and Ethiopian adoptees in Europe and the USA [14-16] but its occurrence in other countries in SSA is reportedly low [17]. Typhoidal *Salmonella* was the second common isolate [9] and a case fatality rate of 15.7% was recorded in hospital admitted children [18]. In 2006, typhoid fever was diagnosed in 37(6.7%) febrile children aged 3-14 years [19]. *S*. Typhimurium is prevalent in SSA [17-19], highly invasive [20-22] and causes high mortality in AIDS patients [23].

Despite the importance of the disease, surveillance and monitoring systems are not in place and the pharmaco-epidemiology of the bacteria is not adequately described. However, integration of previous estimates could provide an insight into the temporal changes and the proportions of drug resistant isolates*.* The objectives of this study were to quantify the temporal changes and estimate the proportions of drug resistant isolates by using meta-analytical methods.

## Methods

The study was conducted according to the guideline of the PRISMA group (Preferred Reporting Items for Systematic Reviews and Meta-Analyses) [24]. The PRISMA check list was used to ensure inclusion of relevant information (Additional file 1). The outcomes of interest were the proportions of ampicillin, co-trimoxazole, chloramphenicol, ceftriaxone, ciprofloxacin and multi-drug resistant isolates. MDR was defined as resistance to three or more drugs.

### Literature search and eligibility criteria

The literature search strategy is described in a previous report [9]. Briefly, studies were searched in Medline, Google scholar and the lists of references of articles. The last search was done on March 30, 2014. To be eligible, a study had to be (i) published, (ii) written in English and (iii) cross sectional or retrospective. Initially, studies with titles and abstracts that are not relevant to the outcomes of interest were excluded. Of the screened articles, duplicates and studies that reported small number of isolates (1-22) were excluded.

### Data extraction

The first author, year of study, location, study design, antimicrobial test methods and interpretative standards, numbers of isolates and numbers of drug resistant isolates were extracted. If the proportion of drug sensitive isolates (q) was reported, the number of resistant isolates was calculated by multiplying the number of isolates (n) by one minus the proportion of drug sensitive isolates (1-q). The study level proportions were derived from the extracted data. The data was abstracted by TG.

### Data analysis

A zero reported for the numbers of drug resistant or sensitive isolates was imputed as 0.5 [25]. The proportions and standard errors were calculated by the following formulae: p = r/n and s. e. =√ p (1-p*)*/n, where r = number of resistant isolates and n = number of isolates. To normalize the distribution of the data, the proportions were transformed to logit event estimates [26,27]: lp = ln [p/(1 - p)], where lp = logit event estimate; ln = natural logarithm; p = study level estimate. The variances were calculated by the following formula: v (lp) = 1/ (np) + 1/[n (1 - p)], where v = variance and n = sample size.

### Bias and heterogeneity analyses

The antimicrobial test methods and the interpretative standards (break point levels) were examined to assess the within study biases. Funnel plots were used to get visual impressions of the across study biases (small study effects). The Begg and Mazumdar adjusted rank correlation and the Egger’s regression asymmetry tests were used to test the significance of funnel plots’ asymmetries.

Heterogeneities were assessed by the Galbraith plot and the Cochran’s Q test. The percentage of the variation attributable to heterogeneity was quantified by the inverse variance index (I^2^) [28]. As the power of the Cochran’s Q test is low in small number of studies, studies were considered heterogeneous if the ratio of Q and the degree of freedom was greater than one. I^2^ values of 25% 50% and 75% were considered as low, moderate and high heterogeneity respectively.

### Temporal change and trend analyses

The average temporal changes were estimated by using a meta-regression model: y = a + bx + u + e; where a = constant, b = regression coefficient, x = year of study, u = error term with known standard deviation and e = error term of the additive component of the variance (tau^2^). If two or more calendar years were reported, the median was considered as year of study. Tau^2^ was estimated by the methods of moments. The Knapp-Hartung variance modification factor was used to calculate the probability values and confidence intervals of the coefficients. The Mantel extension Chi Square for trend was used to assess the changes in the proportion of MDR isolates across decades (1970s/80s, 1990s and 2000s).

### Pooling and sensitivity tests

The DerSimonian and Laird random effects model was used to pool logit event estimates [29]. Pooled logit estimates were back-transformed to proportions by the following formula: p = e^lp^/(e^lp^ + 1), where p = proportion and e = the base of the natural logarithm. Single study omitted influence analyses were done to assess the sensitivities of pooled estimates. A study was considered to be influential if the pooled estimate without it was not within the 95% confidence bounds of the overall mean. The statistical significance of a difference between proportions was assessed by the Yates corrected Chi Square test [30,31]. Alpha was set at 0.05.

Microsoft Office Excel 2007 was used to calculate study level proportions, logit event estimates, standard errors and to back-transform logit event estimates to proportions. Epi info™ (Version 3.5.1, Center for Disease Control, CDC, USA) was used to assess the trend and compare proportions. All other analyses were done by using Stata (Version 11.1, Stata Corp, College Station, Texas).

## Results and discussion

### Literature search and eligible studies

**Figure 1:**
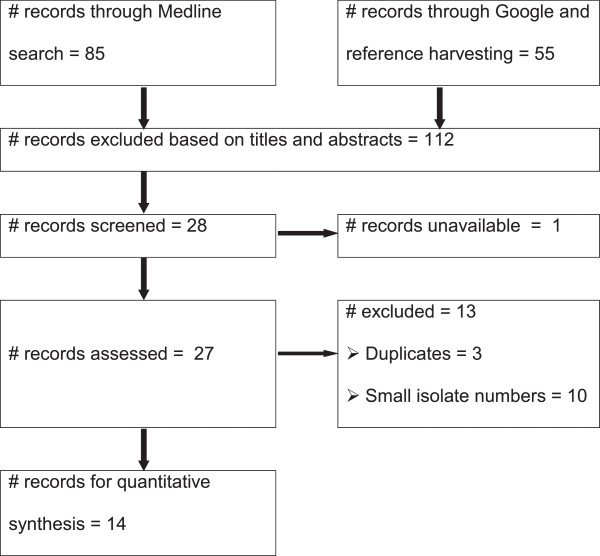
A flow diagram of the selection of eligible studies.

Figure 1 presents the search results. The search yielded 140 studies. The titles and abstracts of 112 studies were not relevant to the outcomes of interest. Of the articles screened for eligibility, 14 were excluded: one study was not available; three were duplicates and ten reported small numbers of isolates. Fourteen studies were eligible for quantitative syntheses. Eleven studies were used to estimate the temporal changes and trends [12,13,32-40]. Five studies [13,37-40] were used to estimate the proportions of drug resistant isolates in the 2000s. Four studies were used to estimate the proportions of drug resistant *S*. Concord in the 2000s [13-15,41]. In the 2000s, only two studies reported the drug resistance features of 15 *S*. Typhi, two *S*. Paratyphi and seven *S*. Typhimurium [13,37]. The search was comprehensive and most, if not all published reports were considered in the analysis. With the exception of one review on *S*. Typhi [42], a review on the antimicrobial resistance features of human *Salmonella* isolates in Ethiopia was not found.

### Characteristics of the eligible studies

The studies were carried out between 1974 and 2009 in Central, Northern, Southern and Eastern Ethiopia (Table 1). Three studies were on S. Concord isolated from Ethiopian adoptees in Europe and the USA. A total of 1030 isolates were tested with a variety of antimicrobials that included the penicillins, cephalosporins, phenicols, quinolones, aminoglycosides, tetracyclines, macrolides and peptides.

**Table 1:** Characteristics of the eligible studies

**Author**	**Location**	**Ys**	**n**	**Number of resistant isolates (%)**					
				**Amp**	**Sxt**	**Chl**	**Cro**	**Cip**	**MDR**
[12]	AA	1974-1981	216	nr	0(0)	nr	nt	nt	39(18.1)
[32]	AA	1975-1980	165	22(13.3)	0(0)	19(11.5)	nt	nt	23(13.9)
[33]^a^	AA	1982-1983	45	12(26.7)	0(0)	8(17.8)	nt	nt	10(22.2)
[34]^b^	AA	1992-1993	37	30(81.1)	28(75.7)	31(83.8)	nt	nt	30(81.1)
[35]	AA	1993-1996	110	40(36.4)	57(51.8)	39(35.5)	nt	nt	41(37.3)
[36]^b^	AA	1995	45	31(68.9)	26(57.8)	21(46.7)	nt	nt	26(57.8)
[37]	JM	2000	59	35(59.3)	24(40.7)	21(35.6)	nt	nt	45(76.3)
[38]^b^	GD	2003-2005	59	54(91.5)	38(64.4)	46(78.0)	nt	6(10.2)	46(78)
[13]^b^	AA/JM	2004-2006	113	93(82.3)	91(80.5)	92(81.4)	89(78.8)	1(0.9)	91(80.5)
[39]	BD	2003-2008	84	77(91.7)	67(79.8)	37(44.1)	nt	nt	72(85.7)
[40]^c^	HR	2007	28	28(100)	nt	18(64.3)	nt	nt	20(71.4)
[41]^d,e^	DM	2007-2009	8	8(100)	8(100)	8(100)	8 (100)	0(0)	8(100)
[14]^d,e,^	EU/USA	2003-2007	35	34(97.1)	35(100)	35(100)	34(97.1)	0(0)	35(100)
[15]^d,e^	BL	2006-2009	26	26 (100)	26(100)	26(100)	26(100)	0(0)	26(100)

AA = Addis Ababa; AA/JM = Addis Ababa and Jimma; Amp = ampicillin; BD = Bahirdar; BL = Belgium; Chl = chloramphenicol; Cip = ciprofloxacin; Cro = ceftriaxone; DM = Denmark; ET = Ethiopia; EU/USA = Europe and the United States of America; HR = Harar; JM = Jimma; GD = Gondar; n = number of isolates; nr = not reported; nt = not tested; Sxt = co-trimoxazole; Ys = Year of study.

^a^The numbers of chloramphenicol and ampicillin resistant isolates were derived from the proportions of sensitive isolates.

^b^The third highest mono-drug resistant isolates number was considered as the number of MDR isolates assuming that the prevalence of MDR isolates is mainly a reflection of the highly resisted drugs.

^c^Data on amoxicillin was substituted to ampicillin assuming that the mechanism of resistance could be similar.

^d^The data show the proportions of drug resistant *S*. Concord.

^e^The exact origin of the adoptees was not reported.

### Risks of bias and heterogeneity

The disk diffusion method was reported in 11 studies [12,13,32-40]. The micro-broth dilution method [14,41] and the Episilon test (E-test) were used to determine minimum inhibitory concentrations (MIC) [13]. Five studies [12,32,33,35,36] used the breakpoint levels of Bauer *et al*. 1966 [43] and nine studies used the standards of NCCLS (National Committee for Clinical and Laboratory Standards) or CLISI (Clinical and Laboratory Standards Institute) [13-15,34,37-41]. The DTU (Technical University of Denmark) food defined resistance break point levels were used to assess resistance to ceftiofur, florfenicol and aminoglycosides [14]. In five studies carried out before 2000, the proportions of ampicillin resistant isolates were underestimated because the breakpoint level (11 mm or less) was lower than the level (13 mm or less) in the modified versions. Similarly, differences in the break point levels of drugs such as tetracycline (14 mm or less in Bauer *et al*. 1966 [43] vs. 11 mm or less in the modified versions) might have affected the proportions of MDR isolates. However, as the occurrence of drug resistant isolates in the earlier years was comparatively lower than in the 2000s, the risks of underestimation or overestimation of the study level proportions are negligible.

**Figure 2:**
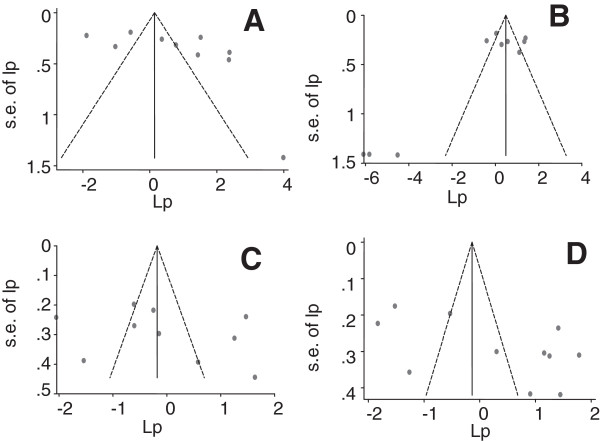
Funnel plots of the logit event estimates (lp): ampicillin (A), co-trimoxazole (B), chloramphenicol (C) and multi-drug (D) resistant isolates.

Figure 2 presents funnel plots of the estimates. The plots demonstrate different patterns. The Egger’s bias coefficients for ampicillin, co-trimoxazole, chloramphenicol and MDR estimates were 7.04 (95%CI = -1.67, 15.76), *p* > 0.05; -4.12 (95% CI = -8.32, 0.08), *p* > 0.05; 4.54 (95% CI = -8.72, 17.81), *p* > 0.05 and 11.06 (95% CI = 0.09, 22.04), *p* > 0.05 respectively and the probability values calculated by the Begg and Mazmudar test were greater than 5%. The plots and tests did not suggest the presence of bias.

**Figure 3:**
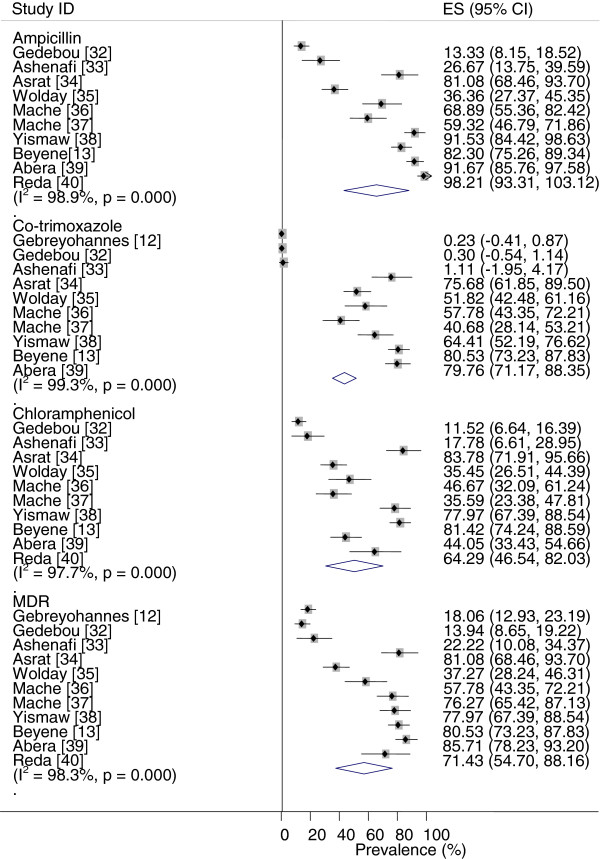
Forest plots of the proportions of drug resistant isolates.

Figure 3 depicts forest plots of the proportions of drug resistant isolates. The percentages of the variations of the logit event estimates attributable to heterogeneities are presented in Tables 2 and 3. The heterogeneities could be mainly due to increases in the proportions of resistant isolates across years.

**Table 2:** Temporal changes of the proportions of drug resistant isolates

**Drug**	**I**^**2 **^**residuals**	**Adjusted R**^**2**^	**b(95% CI)**	***p***
Amp^a^	82.62	77.58	0.54(0.52, 0.55)	0.001
Sxt^b^	72.31	89.52	0.53(0.52, 0.54)	0.000
Chl^a^	84.73	41.05	0.55(0.52, 0.58)	0.003
MDR^b^	88.37	55.32	0.52(0.50, 0.54)	0.021

Amp = ampicillin; b = coefficient of year of study; Chl = chloramphenicol; I^2^ = inverse variance index; MDR = multi-drug resistance; R^2^ = proportion of explained variance; Sxt = co-trimoxazole.

^a^The estimates were derived from data extracted from 10 studies [13,32-40].

^b^The estimates were derived from data extracted from 10 studies [12,13,32-39].

**Table 3:** **Pooled proportions of drug resistant *Salmonella *** **isolated between 2000 and 2008**

**Drug**	**Pooled estimate**		**Heterogeneity**			
	**p(95% CI)**	**Z-*p***	**Q**	**Q-*p***	**Q/df**	**I**^**2**^
Amp	86.01(70.77, 93.98)	0.000	29.32	0.000	7.3	86.4
Sxt	68.01(48.13, 82.98)	0.075	31.54	0.000	10.5	90.5
Chl	62.08(41.05,79.38)	0.258	49.38	0.000	12.4	91.9
Cip	3.61(0.32, 30.62)	0.009	5.7	0.02	5.7	81.5
MDR	79.56(74.90, 83.54)	0.000	3.60	0.464	0.9	0.0

Amp = ampicillin; Chl = chloramphenicol; Cip = ciprofloxacin; df = degree of freedom; I^2^ = inverse variance index; Q = Cochran’s X^2^; Q-*p* = probability value of Cochran’s Q test; Sxt = co-trimoxazole; Z-*p* = probability value of Z test.

### Temporal changes and trend

**Figure 4:**
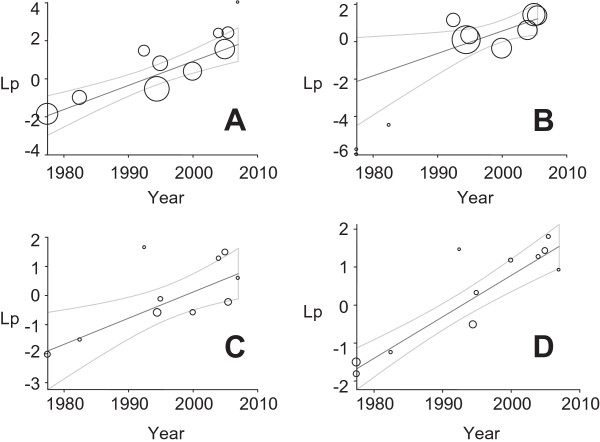
Regression plots of the logit event estimates (lp) against years of studies: ampicillin (A), co-trimoxazole (B), chloramphenicol (C) and multi-drug (D) resistant isolates.

Figure 4 presents regression plots of the logit event estimates of drug resistant isolates against years of studies. The plots demonstrate increasing patterns. The percentages of the explained variances were more than 41% (Table 2). The proportions of MDR isolates differ by decade (X^2^ for trend = 301.82; p < 001). Compared to the 1970/80s, MDR isolates occurred more frequently in the 1990s (OR = 6.92, 95% CI = 4.73, 10.11) and 2000s (OR =18.86, 95% CI = 13.08, 27.19). The pooled proportion of MDR isolates in the 2000s was higher than the proportion in the 1990s [X^2^ = 25.32; p < 0. 001; OR = 2.73 (95% CI = 1.81, 4.1)].

The increase in the proportion of drug resistant isolates could be due to the irrational use of antimicrobials. Several studies have reported the inappropriateness of the prescription and dispensing methods in both the public and private health set-ups. For instance, in Northern Ethiopia, Gondar, ampicillin and penicillin G were two of the three commonly dispensed antimicrobials and most drugs were prescribed by young interns and dispensed by less qualified personnel [44]. In Southern Ethiopia, Hawassa, amoxicillin, ampicillin, chloramphenicol, penicillin G and ceftriaxone were the most commonly prescribed antibacterials [45]. Moreover, the prescriptions had little justifications [46] and the proportion of patients exposed to antimicrobials (>58%) [45-49] was comparatively higher than the standard (20.0%-26.8%) [50]. The pediatric age group was more exposed to antimicrobials than adults and the differences between the prescription behaviors of personnel with shorter and longer pre-service trainings and between public and private health facilities were not significant [47]. Furthermore, prescription-only medications were dispensed without a medical prescription; verbal instructions (87%) were practiced in both pharmacies and rural drug vendors [51] and several patients medicate themselves [52]. Essential drug lists, standard treatment guidelines and drug formulary were available in some but not in all health care settings [53]. In general, the prescription and dispensing practices are not consistent with the rational antimicrobial use guideline and could have favored the selection of antimicrobial resistant microbes.

### Pooled proportions

Table 3 presents the pooled estimates of drug resistant isolates in the 2000s. More than half of the isolates were resistant to ampicillin, co-trimoxazole and chloramphenicol. All single study omitted pooled estimates were within the 95% confidence limits of the respective overall means. The pooled estimates show the magnitude of the problem and the unreliability of ampicillin, co-trimoxazole and chloramphenicol as empirical therapeutic agents. Moreover, the occurrences of isolates resistant to ceftriaxone, 89(78.8%) [13] and norfloxacin, 13(15.5%) [39] were reported.

The higher prevalence of MDR isolates could be associated with the presence of Class I integrons in several isolates. Class I integrons were identified in 52 (53.1%) MDR *Salmonella* predominantly of animal origin [54] and in *S*. Concord with extended spectrum β-lactamase genes (*bla*CTX-M-15) [16]. As data on the genetic features of isolates of human origin is limited, further genetic characterization of isolates is important to understand evolving and epidemic prone strains.

### Dominant serotypes

Table 4 presents pooled proportions of drug resistant *S*. Concord. The MDR profiles are shown in Table 5. Before the 1990s, more than 81% of the isolates were resistant to ampicillin, co-trimoxazole and chloramphenicol [12]. However, in the 2000s more than 97% of the isolates were resistant to the older antimicrobials including the cephalosporins. Furthermore, resistance to aztreonam [14], nalidixic acid [13,15] and intermediate resistance to ciprofloxacin were recorded [14,15,41]. As is the case of *S*. Kentucky [2], *S*. Concord appears to have taken several steps to become pan-resistant and given its higher occurrence and invasiveness (30.6%) [13], it might have caused considerable morbidities and mortalities in Ethiopian children.

**Table 4:** **Pooled proportions of drug resistant *S*. Concord**

**Drug**	**Pooled proportion**^**a**^		**Hetetrogeneity**			
	**p(95% CI)**	**Z-*p***	**Q**	**Q** ***-p***	**Q/df**	**I**^**2**^
Amp	98.68(94.85,99.67)	0.000	0.09	0.954	0.05	0.0
Sxt	98.68(94.85,99.67)	0.000	0.09	0.954	0.05	0.0
Chl	97.98(93.92,99.35)	0.000	0.20	0.903	0.1	0.0
Cro	97.98(93.92,99.35)	0.000	0.20	0.903	0.1	0.0
MDR	98.68(94.85,99.67)	0.000	0.09	0.954	0.05	0.0

Amp = ampicillin; Chl = Chloramphenicol; Cro = ceftriaxone; df = degrees of freedom; Q = Cochran’s X^2^; Q-*p* = probability value of the Cochran’s Q test; Sxt = co-trimoxazole; Z-*p* = probability value of the Z test.

^a^The estimates were based on four studies [13-15,41]. Data from two studies [14,41] were combined before pooling. Intermediate resistance to ciprofloxacin was recorded in twelve isolates [14,41].

**Table 5:** **MDR features of *S*. Concord**

**Author**	**MDR profiles**	**No. (%)**
[13]^a^	Amp Chl Cro Gen Sxt	41(48.8)
	Amp Chl Cro Gen Sxt Tet	29(34.5)
	Amp Chl Cro Gen Nal Sxt Tet	5(6.0)
[14]^b^	Amp Azt Chl (Cep Cfp Cfr Cft Cfz Cpo Cro Ctz ) Str SulTmp	34(97.1)
	Amp Azt Chl (Cep Cfp Cfr Cft Cfz Cpo Ctz ) Str SulTmp	1(2.9)
[15]^c^	Amp Chl Cro Gen Str Sul Sxt Tmp	3(11.5)
	Amp Chl Cro Gen Sul Str Sxt Tet Tmp	19(73.1)
	Amp Chl Cro Gen Nal Str Sul Sxt Tet Tmp	4(15.4)
[41]^d^	Amp Chl Cfo Cro Gen Str Sul Tet Tmp	8 (100)

Amp = ampicillin; Azt = aztreonam; Cep = cephalothin; Cfo = cefotaxime; Cfp = cefepime; Cfr = cefuroxime; Cft = ceftiofur; Cfz = cefazolin; Cpo = cefpodoxime; Cro = ceftriaxone; Ctz = ceftazidime; Chl = chloramphenicol; Gen = gentamicin; Nal = nalidixic acid; Sul = sulfamethoxazole; Str = streptomycin; Sxt = co-trimoxazole; Tet = tetracycline; Tmp = trimethoprim.

^a^Each profile was recorded in more than 5% of the MDR isolates. One isolate was resistant to ofloxacin.

^b^Resistance to gentamicin (97%), tetracycline (69%) and intermediate resistance to ciprofloxacin (14%) were recorded.

^c^Seven isolates showed intermediate resistance to ciprofloxacin.

^d^Resistance to florfenicol (6/8), colistin (1/8) and intermediate resistance to ciprofloxacin (6/8) were recorded.

Data on the sensitivities of *S*. Typhi, *S*. Paratyphi and *S*. Typhimurium are limited. However, there are evidences on the occurrence of isolates that are resistant to the older drugs [13,37] and norfloxacin [55]. In addition, *S*. Typhimurium isolates of animal origin were shown to be resistant to several drugs including ceftiofur and ciprofloxacin [56-61]. Furthermore, MDR genes located on a virulence-associated plasmid of *S*. Typhimurium were identified [62] and ST313 appears to have occupied a niche provided by HIV, malaria, and malnutrition in SSA [63].

### Implications and limitations

The results of this study have several implications in clinical practices and in policy and research issues. The comparatively lower proportion of ciprofloxacin resistant isolates suggests the potential use of ciprofloxacin as an empirical therapeutic agent. However, as there are evidences of intermediate resistance to ciprofloxacin, alternative drugs should be included in the essential drug list of the country so as to manage severe and life threatening infections. The fluoroquinolones were used to treat children suffering from MDR Gram negative bacterial infections [64] and azithromycin is an attractive alternative against MDR *Salmonella*[65,66]. An association between mass oral azithromycin treatment and a reduction in all-cause and infectious mortalities in rural children was recorded [67].

Policy and decision makers could make use of the evidences as inputs to re-enforce the drug use policy and to devise strategies and measures that could help reduce the rates of emergence of drug resistant pathogens. Apart from the active involvement of the regulatory bodies and the long-arm of the law on drug smuggling and over- the-counter sells of prescription-only drugs, educational initiatives could be of practical significance to reduce the rates of emergence of drug resistant pathogens in the country. Educational programs were reported to be effective in improving the diagnostic qualities of health workers and reducing unjustified prescriptions [68]. Although information sources offer a framework to base educational intervention measures, a regular training is more effective than guidelines alone [69].

The reservoirs and host ranges of the NTS isolates are unknown and the factors associated with the emergence of drug resistant strains are not adequately described. Some of the strains (e.g. *S*. Concord) are becoming international concerns and containment of the problem needs an international approach [15]. To this effect, a large scale investigation into the pharmaco-epidemiology of *Salmonella* is needed and research efforts should be directed towards hypothesis driven preventive measures.

Apart from the small number of eligible studies, the exact origins of the study subjects were not reported. The pooled estimates were also derived from data collected between 2000 and 2009. Therefore, as most patients could be from the urban areas where access to health care facilities is relatively better than the rural areas, the estimates are more applicable to the urban than the rural population and the current proportions of drug resistant isolates may be higher than the present estimates.

## Conclusion

The proportion of drug resistant *Salmonella* has increased since the 1970s and a considerable proportion of the isolates are multi-drug resistant. Ciprofloxacin could be used as an empirical therapeutic agent. The third generation cephalosporins are not useful against *S*. Concord infections. Alternative drugs should be included in the essential drug list and intervention measures should be taken to re-enforce the drug use policy. Further large scale studies are required to describe the pharmaco-epidemiology of *Salmonella* in Ethiopia.

## Competing interests

The author declares no competing interests.

## Author’s contribution

TG conceived the design, searched the literature, extracted and analyzed the data, interpreted the results and drafted the manuscript.

## Pre-publication history

The pre-publication history for this paper can be accessed here:

http://www.biomedcentral.com/2050-6511/15/51/prepub

## Supplementary Material

Additional file 1 PRISMA Checklist. Click here for file
